# Supplementary material for: Molecular Determinants for OMF Selectivity in Tripartite RND Multidrug Efflux Systems
Source: Antibiotics (Basel). 2022 Jan 18;11(2):126. doi: 10.3390/antibiotics11020126 (PMC8868134; doi:10.3390/antibiotics11020126)
Supplement: Supplementary file 1 [file antibiotics-11-00126-s001.zip › antibiotics-1554761-supplementary.pdf]

Supplementary Materials

Molecular Determinants for OMF Selectivity in Tripartite RND Multidrug Efflux Systems

Esther Boyer, Jean Dessolin, Margaux Lustig, Marion Decossas, Gilles Phan, Quentin Cece, Grégory Durand, Véronique Dubois, Joris Sansen, Jean-Christophe Taveau, Isabelle Broutin, Laetitia Daury and Olivier Lambert

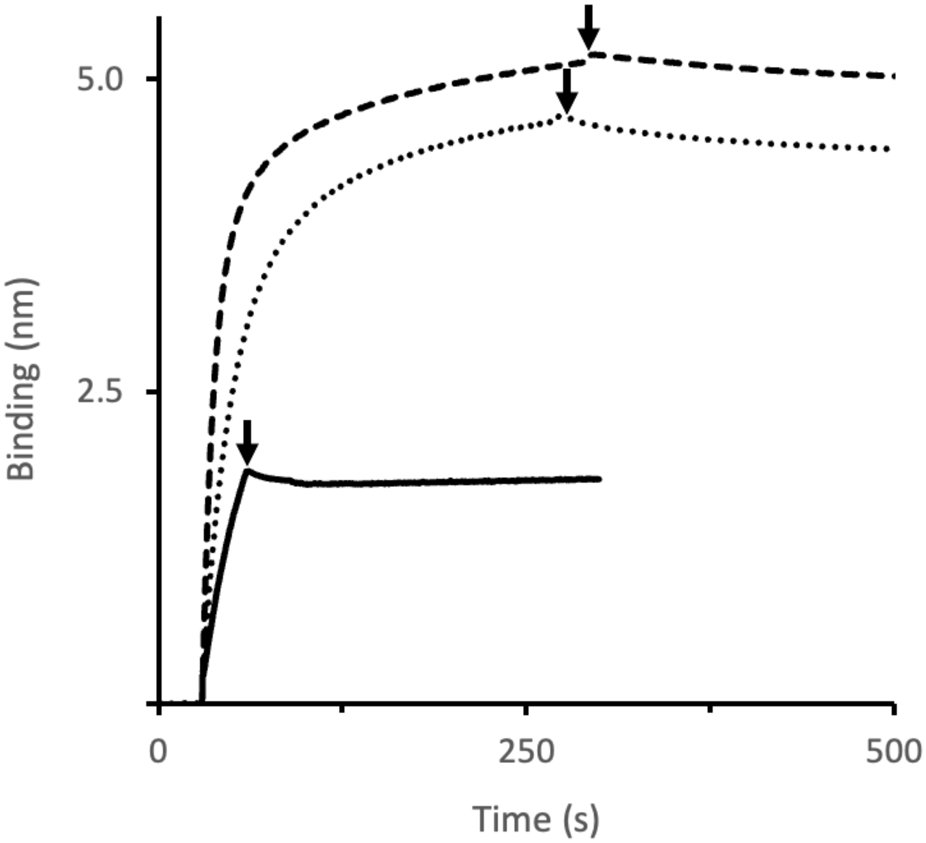

Figure S1. Association/dissociation curves for BNAPol-OprM loading onto the streptavidin biosensor. Different conditions for loading OprM onto the biosensor have been tested before starting experiments for all the OMFs tested: 46 μg.mL^{-1} - 5 min (dashed line); 23 μg.mL^{-1} - 4 min (dotted line); 15 μg.mL^{-1} - 30 s (solid line). Each was followed by a 4 min dissociation step (indicated by the black arrows). For experiment described in Figure 2, the non-saturated condition corresponding to a loading with 15 μg.mL^{-1} OMF for 30 s was chosen for preserving MexA accessibility towards OMF.

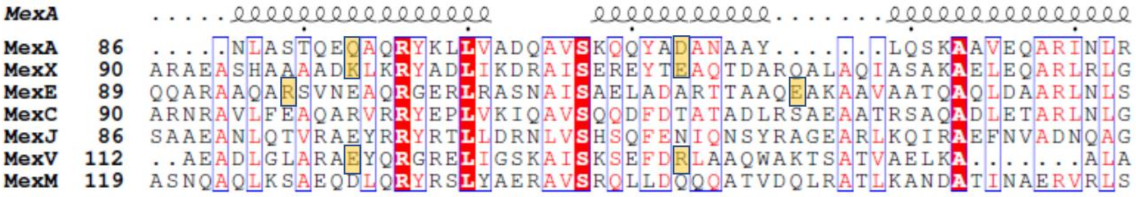

Figure S2. Alignment of primary sequences of PAP α-helix hairpin domains from *P. aeruginosa*. MexX presents putative pair anionic cationic residues as MexAQ93R, and MexV as well (orange square). For MexE, another paired residue could exist.

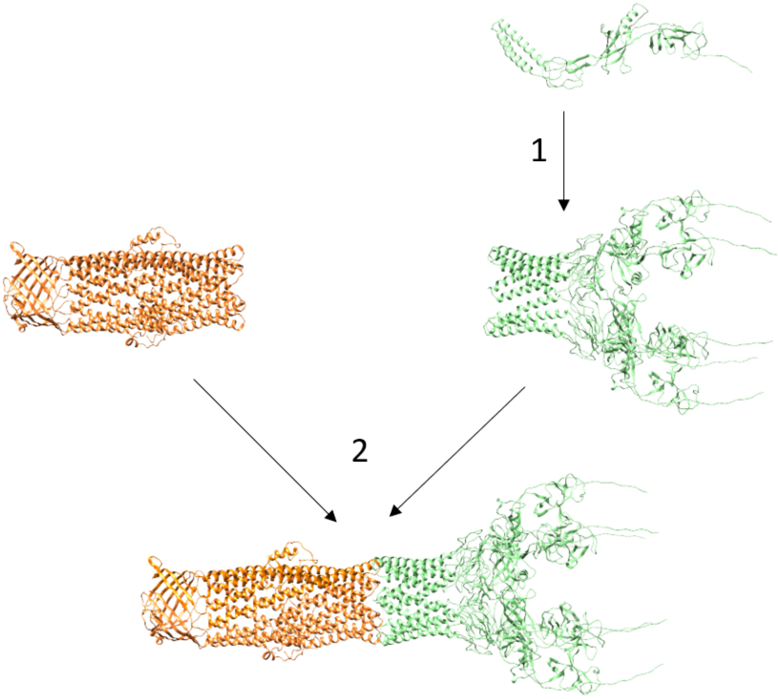

Figure S3. Model simulation of PAP hexamer and OMF-PAP complex. In a first step, a hexamer of PAP (i.e. $MexA_{Q93R}$, MexX or MexE) was generated from a model of a PAP monomer using SymmDock. The second step consists in generating an OMF-PAP complex using PatchDock. OprM, OprN and TolC were assembled with $MexA_{wt}$ and $MexA_{Q93R}$. Binding energy was estimated by FireDock (Table S3).

Table S1. Details about average images from 2D classification of tripartite complexes and $MexA_{Q93R}$.

	Total particles	Class number	Selected classes
MexA-MexB-OprM	4,480	100	48; 40; 27; 40
$MexA_{Q93R}$-MexB-OprM	46,145	50	379; 424; 353; 443
$MexA_{Q93R}$-MexB-OprN	1,236	30	37; 40; 52; 59
$MexA_{Q93R}$-MexB-TolC	1,191	60	23; 22; 19; 14
$MexA_{Q93R}$-MexB- $OprM_{\Delta 473-485}$	14,025	150	111; 112; 114; 123
$MexA_{Q93R}$	11,572	50	228

Total particles corresponding to the particle set submitted to 2D classification. Class number corresponding to the number of obtained classes. Selected classes corresponding to the number of particles from each class shown in Figure 5 (number sequence follows alphabetical letter in Figure. 5). For $MexA_{Q93R}$, number corresponding to average image shown in Figure. 3 inset.

Table S2. Hexamer assembly of MexA and variant modelled by SymmDock.

	MexA	$MexA_{Q93R}$	$MexA_{Q93A}$	$MexA_{D113A}$	$MexA_{Q93R+D113A}$
Score	17540	17710	17190	17556	17492

Monomers of MexA were submitted to C6 symmetrical docking with SymmDock server. The score of $MexA_{Q93R}$ was higher than $MexA_{wt,}$ indicating a better stabilisation probably due to the interaction between R93 from one protomer and D113 from the adjacent protomer. $MexA_{Q93A}$ showed a lower score than that $MexA_{wt}$. $MexA_{D113A}$ and $MexA_{Q93R+D113A}$ showed scores similar to $MexA_{wt}$ score. Superimposition of these symmetrical dockings led to geometrically identical assemblies very close to $MexA_{wt}$ (PDB: 6TA5).

Table S3. Scoring of OMF-MexA interaction.

	OprM-MexA	OprM-$MexA_{Q93R}$	OprN-MexA	OprN-$MexA_{Q93R}$	TolC-MexA	TolC-$MexA_{Q93R}$
FireDock	−76.83	−105.90	−40.28	−40.23	−74.00	−97.90

OprM (6TA5), TolC (5NG5) and OprN (modified from 5IUY to generate an open conformation) in the presence of C6 symmetrized MexA-wild type or mutant were then submitted to PatchDock protein-protein docking. After protein-protein docking, FireDock algorithm allowed a re-scoring of the obtained complexes. The higher scores obtained for OprM-MexA than for OprN-MexA and TolC-MexA likely suggested that the tip-to-tip contact of cognate partners are more suitable than with non-cognate partners. Refinement with FireDock provided evidence that a stabilised $MexA_{Q93R}$ hexamer improves the score of the OMF-PAP complex.
